# Computed tomography validated right ventricular mid‐septal lead implantation using right ventricular angiography

**DOI:** 10.1002/joa3.12591

**Published:** 2021-07-11

**Authors:** Jayaprakash Shenthar, Maneesh K. Rai, Siva S. Chakali, Vivek Pillai, Tammo Delhaas

**Affiliations:** ^1^ Electrophysiology Unit Department of Cardiology Sri Jayadeva Institute of Cardiovascular Sciences and Research Bangalore India; ^2^ Department of Biomedical Engineering Maastricht UMC+ Maastricht The Netherlands

**Keywords:** alternate site pacing, cardiac pacing, mid‐septal pacing, pacemaker implantation septal pacing

## Abstract

**Background:**

Right ventricular (RV) mid‐septal pacing has been proposed as an alternative to RV apical pacing. Fluoroscopic and electrocardiogram criteria are unreliable for predicting the RV mid‐septal lead position. This study aimed to define the optimal RV mid‐septal pacing site using RV angiography.

**Methods:**

We randomized patients undergoing pacemaker implantation (PPM) to the RV angiography‐guided group (Group A) or conventional fluoroscopy‐guided group (Group F). In Group A, we performed an angiogram in right anterior oblique (RAO 30°), left anterior oblique (LAO 40°), and left lateral (LL) views. We made a 5‐segment grid in RAO 30° and LL views and a 3‐segment grid in LAO 40° on the angiographic silhouette to define the lead position. Computed tomography (CT) was used to validate the lead tip position in both groups.

**Results:**

We enrolled 53 patients (Group A: 26, Group F: 27) with a mean age of 55.9 ± 12.2 years. CT images validated the lead position in the mid‐septum (Group A, 23 [88.5%]; Group F, 11 [40.7%], *P* = .0003) and anteroseptal (Group A, 3 [11.5%]; Group F, 5 [18.5%], *P* = .24). In Group F, the lead was in the anterior wall in 9 patients (33.3%) and the right ventricular outflow tract in 2 (7.4%) patients and none in these two positions in Group A. The lead tip in segment one on the angiographic 5‐segment grid in RAO 30° and LL views indicated a mid‐septal lead position on CT.

**Conclusions:**

RV angiography is safe and may be used to confirm the mid‐septal lead position during PPM.

## Graphical abstract

The appropriate site for mid‐septal pacing is not defined. This study aimed to identify the site of mid‐septal implantation as guided by right ventricular angiography and was validated by computed tomography.

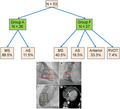

## 1 INTRODUCTION

Right ventricular (RV) apical (RVA) pacing causes abnormal electrical and mechanical activation, resulting in alterations in cardiac metabolism, perfusion defects, and remodeling, leading to left ventricular dysfunction.^1^ To prevent the harmful effects of RVA pacing, various nonapical sites, such as the right ventricular outflow tract (RVOT) and right ventricular upper/mid‐septum, and para‐Hisian pacing, have been proposed.^2^, ^3^, ^4^ Though physiological pacing of His bundle and the left bundle is growing in popularity, it has the disadvantages of a steep learning curve, availability of the hardware from a single vendor, the necessity of an electrophysiology recording system, and knowledge of the conduction system’s anatomy limiting it to specialized centers. A meta‐analysis of several studies comparing RV apical to nonapical sites showed mixed medium and long‐term effects on left ventricular function.^5^ The most significant limitation of the studies on nonapical RV pacing is the nonuniformity of the area of ventricular septal stimulation, along with a lack of accuracy of the septal lead placement. Studies on mid‐septal pacing have used fluoroscopic criteria for the lead tip position in the left anterior oblique (LAO) 40°^6^ and the right anterior oblique (RAO) 30° views.^7^ A negative QRS complex or a q wave in lead I on the surface electrocardiogram (ECG) is supposed to indicate RV septal pacing.^8^, ^9^, ^10^ Three‐dimensional (3D) echocardiography and computed tomography (CT) scans have shown the fluoroscopic and ECG criteria are inaccurate for predicting the mid‐septal lead position.^11^, ^12^, ^13^ Placement of RV lead in the mid‐septum using fluoroscopy alone is challenging because of the interventricular septum's complex anatomy and the inability to define the mid‐septal area. Angiography has been suggested as an additional imaging technique during permanent pacemaker implantation (PPM) to determine the septal anatomy in real‐time and improve mid‐septal lead implantation accuracy.^12^, ^14^, ^15^

The study aimed to define the optimal site for RV mid‐septal lead implantation using contrast RV angiography. We compared the contrast angiography‐guided mid‐septal lead implantation with the previously described fluoroscopic‐guided lead implantation technique ^6^, ^7^ using computed tomography.

## 2 METHODS

The present study is a single‐center, randomized study conducted in a tertiary care teaching hospital. We enrolled adult patients (age 21‐80 years) with symptomatic atrioventricular (AV) block, in sinus rhythm, with normal left ventricular function (Echo LVEF >55%), and no contraindication for contrast agents. Patients were excluded based on the following criteria: baseline LV ejection fraction (LVEF) ≤ 55%, presence of atrial arrhythmias, renal dysfunction (Serum creatinine >1.3 mg/L), aged <21 or >80 years, current pregnancy, history of contrast allergy, or unwillingness to participate. Patients with atrial fibrillation not requiring a dual‐chamber pacemaker and sick sinus syndrome with normal AV conduction were also excluded from the study. Patients who gave written informed consent were prospectively enrolled in the study. All patients underwent detailed clinical, electrocardiographic, biochemical, and two‐dimensional echocardiographic evaluations before the procedure. A research nurse randomized the patients to angiography‐guided (Group A) or conventional fluoroscopic‐guided (Group F) pacemaker implantation. The institutional clinical research and ethics committee approved the study.

Pacemaker implantation was performed after 6 hours of fasting by the patient. Intravenous normal saline was started at 60 mL/h from 6 hours before the procedure. We performed blood urea and serum creatinine estimations before and 24 hours after the patients’ procedure. During the implant procedure, two separate venous accesses were obtained by extrathoracic axillary vein puncture. All patients received a dual‐chamber pacemaker using a standard 58 cm, 7‐French, bipolar, steroid‐eluting, active‐fixation lead (Medtronic CapSureFix Novus 5076, Medtronic Inc., Minneapolis, MN, USA) for the right ventricle and a 52 cm bipolar, active fixation lead (Medtronic CapSureFix Novus 5076, Medtronic Inc) for the right atrium.

In Group A, two sheaths were introduced over the guidewire, with one sheath being a 7F peel‐away introducer and the other a 7F angiographic sheath with a sidearm (AVANTIR+, Cordis Corporation, Santa Clara, CA, USA). The active fixation pacing lead was introduced through the peel‐away introducer and advanced into the right atrium using a straight stylet. A 7F angled pigtail catheter was advanced through the angiographic sheath and into the right ventricle over a 0.035” Teflon guidewire and positioned in the mid‐RV. The lead was then advanced into the pulmonary artery using a manually shaped two‐dimensional (2D) stylet. The 2D stylet was then exchanged for a manually fashioned dual‐curve 3D stylet as described previously.^16^ Using the posterior–anterior (PA) or RAO 30° fluoroscopic views, the lead with a 3D stylet was withdrawn using a gentle counterclockwise torque until the lead tip fell below the RVOT with an abrupt leftward movement. The lead was then gently advanced, maintaining the torque to direct the tip to the mid‐septum. Before deploying the screw, we performed RV angiography with the lead tip in place using the pressure injector. Angiography was done by injecting 30 mL of nonionic contrast Iohexol (Omnipaque 350; GE Healthcare, Oslo, Norway) at 15 mL/s and 700 PSI, and cine films were recorded at 15 frames/s. RV angiogram was performed in the RAO 30°, LAO 40°, and left lateral (LL) 90° views. The amount of contrast used was restricted to <5 mL/kg body weight or a maximum dose of 300 mL.^7^

**FIGURE 1:**
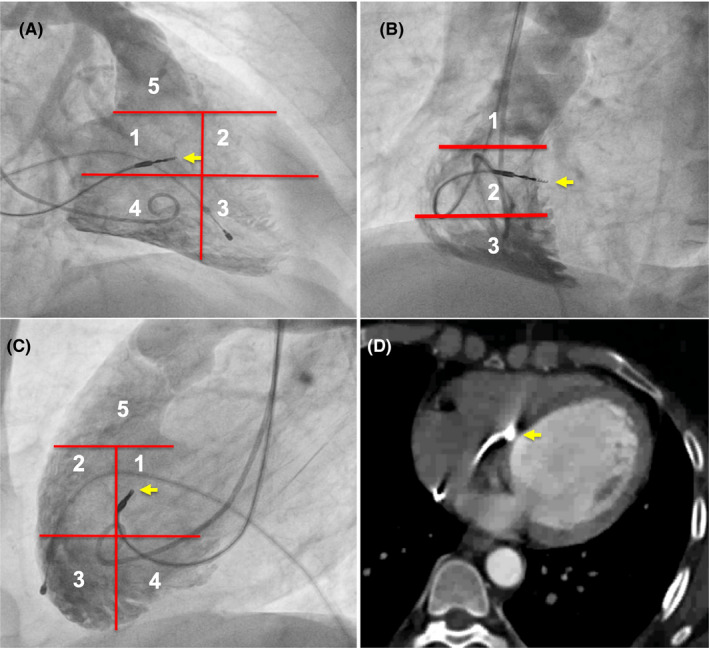
Right ventricular angiographic and CT scan of the mid‐septal lead position. RV angiography (A‐C) along with the described segments and the comparative CT scan (D) in diastole. Panel demonstrates (A) RAO 30° with 5 segments, (B) LAO 40° with 3 segments, (C) left lateral with five segments, and (D) CT scan. Note the tip of the ventricular lead (yellow arrow) in segment 1 in RAO 30° and LL views and the middle segment in the LAO 40° view in panels (A‐C), and the lead tip in the mid‐septal region on the CT scan. LAO, left anterior oblique view; LL, left lateral 90° view; RAO, right anterior oblique view; RV, right ventricle

**FIGURE 2:**
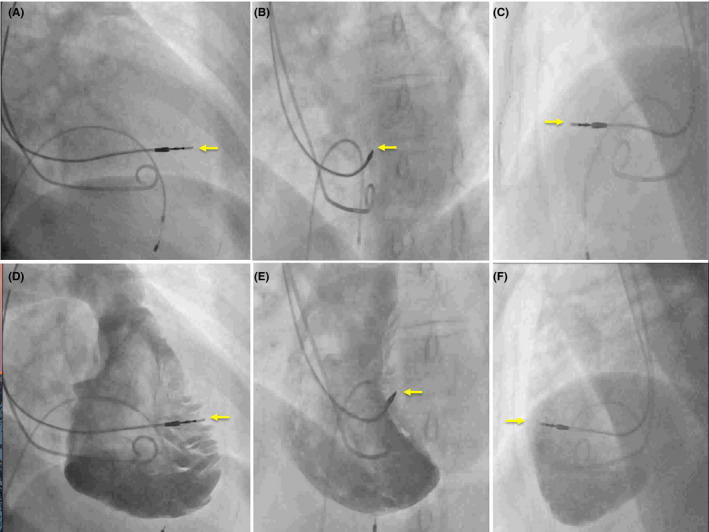
Fluoroscopy and RV angiography of the lead tip in the anterior wall. The top panel demonstrates fluoroscopy in (A) RAO 30°, (B) LAO 40°, and (C) LL, the lower panels (D‐F) show the RV angiography in similar views. The lead tip (yellow arrow) on fluoroscopy appears to be in the middle of the cardiac silhouette in RAO and LAO views on fluoroscopy, but the angiogram demonstrates that the lead tip is in the anterior wall. LAO, left anterior oblique view; LL, left lateral 90° view; RAO, right anterior oblique view; RV: right ventricle

To assess and define the lead tip’s fluoroscopic position, diastolic frames of the RV angiography in RAO 30°, LAO 40°, and left lateral views were used. In the RAO 30° view, the angiographic cavity image was divided into five segments by two horizontal lines and one vertical line, as shown in Figure 1A‐C. The first horizontal line was from the top hinge of the tricuspid valve to the border of the cardiac silhouette. The second horizontal line was from the middle of the tricuspid valve to the cardiac silhouette border. One vertical line through the center of the lower horizontal line created a 5‐segment grid. In the left lateral views, two horizontal lines divided the RV cavity into five segments. The first horizontal line from the top hinge of the tricuspid valve to the border of the cardiac silhouette, the second from the lower hinge of the tricuspid valve to the edge of the cardiac silhouette. The vertical line was drawn through the middle of the lower horizontal line to create the 5‐segment grid. In the LAO 40° view, the RV angiographic cavity was divided into three segments by two horizontal lines, one drawn from the top of the tricuspid valve and the second drawn halfway between the first line to the apex dividing the RV into three segments. The lead tip was targeted to segment 1 in the RAO 30° and LL views and the midsegment in the LAO 40° view. If the lead was not in the desired position (Figure 2), it was repositioned using the RV angiogram as a road map. If the position was in doubt, a repeat angiogram was performed to confirm the final lead position. After achieving a satisfactory ventricular lead position, the screw was deployed, pacing parameters checked, and the lead stability was assessed with coughing and deep breathing. The angiographic sheath was exchanged for a peel‐away introducer, and the atrial lead was implanted using the standard technique.

In Group F, the pacing lead was advanced to the pulmonary artery using a 2D stylet in the PA projection. The 2D stylet was exchanged for a manually fashioned 3D stylet, and the lead retracted into the mid‐septum as described above using the defined fluoroscopic views. The screw was deployed once the fluoroscopic views indicated the mid‐septal lead position in the LAO 40° and RAO 30° views. As described previously in the literature, the lead tip was considered in the mid‐septum if, in the LAO 40° view, the lead tip faced the spine with an angulation between the horizontal plane and the axis of the distal part of the lead between 0° and 60°. In the RAO 30° view, the lead tip was in the middle quadrant.^3^, ^7^, ^17^

A 12‐lead ECG was recorded at the end of the procedure using multichannel electrophysiology (EP) analysis system (EP tracer, Schwarzer Cardiotek GmbH, Heilbronn, Germany). The surface ECG recording was filtered at 0.05‐150 Hz, 10 mm/1 mV amplitude, and measurements were recorded at a sweep speed of 100 mm/s. The ECG parameters were analyzed on the 12‐lead ECG by an independent operator and included QRS duration, QRS axis, presence or absence of QRS notching in limb leads, presence or absence of a q wave, or negative QRS complex in the lead I, and a QRS transition zone in the precordial leads.

A cardiac CT scan was performed after the implantation during the outpatient follow‐up. A dual‐source, 64‐slice Siemens Definition Flash CT scanner (Siemens, Erlangen, Germany) was used to acquire images with a tube voltage between 100 and 120 kV and a tube current of 200‐300 mA, depending on patient size. While performing the CT scan, the pacemaker was programmed to VVI 60 bpm to reduce the motion artifact. Scans were performed after 75 mL of Omnipaque 350 was injected at 6 mL/s, followed by 50 mL of saline. During image acquisition, prospective ECG gating was done using a phase window (70%‐80%) and an image matrix of 512 × 512 pixels, and initial reconstruction was performed at the 75% phase (0.75‐mm slice thickness and 0.5 mm intervals) using B26 (soft) and B46 (hard) kernels. Offline analysis was performed with data sets that were transferred to an external workstation. For accurate localization of the RV lead axial slices, oblique reconstructions and maximum‐intensity projection images were used. Two experienced radiologists blinded to the other’s results performed the analysis, and any disagreement between the two was resolved by consensus. RV lead positioning was defined in short‐ and long‐axis planes as designated by Pang et al.^12^ The RV lead tip positions were divided into mid‐septum, anteroseptal junction, anterior, and free wall. The fluoroscopic and angiographic lead tip positions were compared and validated using CT scan images.

The primary endpoint was an accurate RV mid‐septal lead implant validated by CT scan. The secondary endpoint was a narrower QRS on a 12‐lead ECG compared with nonmid‐septal sites with no complications during or after the procedures.

Continuous values are expressed as mean ± SD. Categorical data are expressed as numbers or percentages. Continuous data were analyzed using the unpaired Student’s *t* test. Fisher’s exact test was used for evaluating dichotomous variables. A *P*‐value <.05 was considered statistically significant. Statistical analyses were performed using SPSS software version 22 (SPSS, Inc.).

## 3 RESULTS

A total of 57 patients were initially enrolled in the study. Four patients did not consent for a follow‐up CT scan and were not considered in the final analysis. For the final analysis, 53 patients remained enrolled, with 26 in Group A and 27 in Group F. The details of the clinical, echocardiographic, and other relevant characteristics of the patients are summarized in Table 1. The patients’ mean age was 55.9 ± 12.2 years (range 26‐75 years), and there were 33 men and 20 women. The total amount of contrast used in Group A was 143 ± 36 mL. The mean serum creatinine levels changed from 1.0 ± 0.2 to 1.1 ± 0.3 mg/dl (*P* = .08) in Group A, and from 1.0 ± 0.1 to 1.0 ± 0.2 mg/dl (*P* = .5) in Group F, neither of which were statistically significant changes. Serum creatinine was not elevated above 1.5 mg/dl in any of the patients. There was no significant difference in the increase of the serum creatinine levels between Group A and Group F (1.1 ± 0.3 vs 1.0 ± 0.2 mg/dl, *P* = .81) postprocedure. Except for the lead dislodging in one Group F patient (repositioned uneventfully after 2 weeks), there were no complications related to the procedure. The fluoroscopy time was 7.3 ± 2.6 min in Group A and 4.8 ± 3.2 min in Group F (*P* < .001).

**TABLE 1:** Clinical, echocardiographic, and implant parameters (N = 53)

Parameter	Group A (N = 26)	Group F (N = 27)	*P* value
Age (years) (mean + SD)	57.6 + 8.5	58.4 + 7.3	.29
Male: female	15:11	18:9	.20
LVIDD (mm) (mean + SD)	51.9 + 5.8	52.1 + 4.7	.44
LVEF (%) (mean + SD)	59.2 + 4.8	58.4 + 6.5	.31
Indication:			
2° AVB	2	3	.33
High grade	8	6	.24
Complete AVB	16	18	.34
Serum creatinine mg/dl (mean + SD)	1.0 + 0.2	1.0 + 0.1	.50
Fluoroscopy (min) (mean + SD)	7.3 + 2.6	4.8 + 3.2	.001
QRS amplitude (mV) (mean + SD)	9.7 + 4.1	10.4 + 3.6	.25
RV threshold (V) (mean + SD)	0.71 + 0.15	0.69 + 0.18	.33
RV impedance (Ω) (mean + SD)	678 + 144	663 + 138	.35

Abbreviations: AVB, atrioventricular block; LVEF, left ventricular ejection fraction; LVIDD, left ventricular internal diameter in diastole; RV, right ventricle.

Assessment of the RV angiography in Group A at the time of implantation revealed that in 15 patients, the lead tip was not in the mid‐septum in the first attempt. Of the 15 leads, seven were in the anterior wall, five were in the anteroseptal junction, and three were in the RVOT. In all 15 patients, the lead was repositioned to the mid‐septum using a smaller secondary curve to reach the target zone since the larger curve tended to direct the lead tip either to the RVOT or the anterior wall region.

**FIGURE 3:**
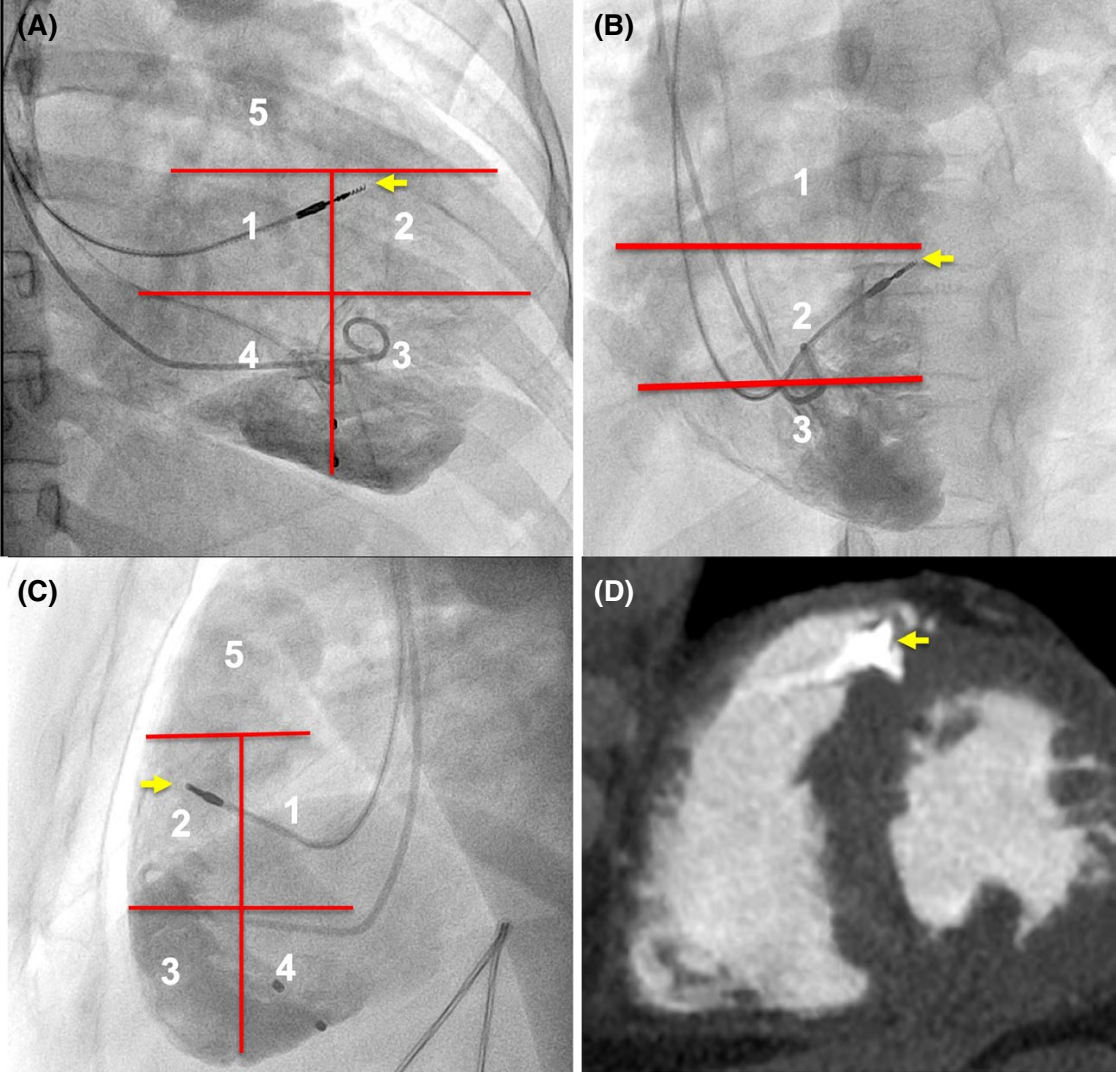
Right ventricular angiographic and CT scan of the anteroseptal lead position. RV angiography (A‐C) along with the described segments and the comparative CT scan (D) in diastole. Panel demonstrates (A) RAO 30° with 5 segments, (B) LAO 40° with 3 segments, (C) left lateral with 5 segments, and (D) CT scan. Note the tip of the ventricular lead (yellow arrow) in segment 2 in RAO 30° and LL views and the upper part of the middle segment in the LAO 40° view in panels (A‐C). The CT scan in the short axis view in (D) shows the lead tip in the anteroseptal region. LAO, left anterior oblique view; LL, left lateral 90° view; RAO, right anterior oblique view; RV, right ventricle

The CT comparison of the lead tip position between the two groups is presented in Table 2. In Group A, the lead tip was in the mid‐septal location in the 5‐segment grid if the tip was in segment 1 in the RAO 30° and LL views and the midsegment in the LAO 40° view (Figure 1A‐C). Figure 1D shows the lead tip anchored to the mid‐septum on CT. The lead tip was in the anteroseptal junction on CT if the lead tip in the 5‐segment grid was in the proximal half of segment 2 in the RAO 30° and LL views and the midsegment in the LAO 40° view (Figure 3A‐D). It was in the anterior wall on CT in the distal half of segment 2 in the RAO 30° and LL views. The lead tip in segment 5 in the RAO 30° and LL views and the upper segment in the LAO 40° view indicated an RVOT location on the CT image.

**TABLE 2:** Computed tomography comparison of the position of the lead tip in both groups

Parameters	Group A (n = 26)	Group F (n = 2)	*P*‐value
Mid‐septum, n (%)	23 (88.3)	11 (40.7)	<.001
Anteroseptal junction, n (%)	3 (11.5)	5 (18.3)	.24
Anterior wall, n (%)	0 (0)	9 (33.3)	<.05
RVOT, n (%)	0 (0)	2 (7.4)	<.05

Abbreviation: RVOT, right ventricular outflow tract.

ECG characteristics of the two groups are shown in Table 3. Group A patients had significantly narrower QRS complexes, lesser q waves in the lead I, a more leftward axis, and earlier QRS transitions in the chest leads. Patients in Group F had significantly more q waves in the lead I, a more rightward axis, and a later QRS transition on chest leads. There was no significant difference in the notching of Q waves in the inferior leads.

**TABLE 3:** Paced electrocardiographic characteristics

Parameter	Group A (n = 26)	Group F (n = 27)	*P*‐value
QRS duration (ms), mean ± SD	134.5 ± 18.2	148.8 ± 22.3	.007
Q in lead I, n (%)	4 (15.4)	19 (70.3)	<.001
QRS amplitude in I (mV), mean ± SD	5.8 ± 4.1	1.4 ± 2.2	<.001
QRS amplitude in II (mV), mean ± SD	6.7 ± 3.5	6.1 ± 5.2	.31
QRS amplitude in III (mV), mean ± SD	1.2 ± 5.2	4.4 ± 4.3	.01
Notching in inferior leads (n, %)	10 (38.5)	15 (55.6)	.10
QRS axis (°), mean ± SD	19.7 ± 36.7	68.6 ± 40.2	<.001
QRS transition in chest leads, mean ± SD	4.4 ± 1.1	5.1 ± 1.8	.047

Abbreviation: SD, standard deviation.

## 4 DISCUSSION

This study makes a novel contribution to the literature by defining specific areas of the RV septum, and especially the mid‐septal area, using real‐time angiography, corroborated by CT scans, as opposed to conventional fluoroscopy. The study’s principal findings are as follows: first, the use of right ventricular angiography helps delineate the RV anatomy during PPM and helps to target the mid‐septal area accurately, as validated by a CT scan. Second, the use of conventional fluoroscopy as a guide for implanting the lead in the mid‐septal position leads to an inaccurate mid‐septal lead position in >50% of cases. Third, mid‐septal lead implantation results in a narrower paced QRS complex compared with the nonmid‐septal lead implant. Fourth, pacing from the mid‐septum results in narrower QRS complexes, lesser q waves in the lead I, a more leftward axis, and earlier QRS transitions in the chest leads.

In defining selective nonapical pacing sites, the literature has traditionally defined anatomic positions broadly. Since the RV has a complex anatomy, it is difficult to visualize and verify lead implant sites outside the traditional RV apical position using fluoroscopy alone.^9^

Mǎrgulescu et al. using 3D echocardiography noted that fluoroscopic and ECG criteria were not very accurate in differentiating RV apical, mid‐septal, mid‐free wall, and RVOT pacing sites.^11^ Domenichini et al. conducted a randomized study that used 2D echocardiography to locate the lead position. They found that the correct mid‐septal lead position was observed in only 54% of patients, with the rest being in the anterior wall.^18^ In a nonrandomized study by Ng et al. using 2D echocardiography to validate the LAO 40° criteria, the RV septal pacing site was heterogeneous, and ECG and chest x‐rays were in only modest agreement with echocardiographic data.^19^ It is now evident that CT scanning is more reliable and accurate to identify the RV septal lead position than echocardiography or magnetic resonance imaging.^13^

The original ECG criteria for RV septal pacing using fluoroscopic landmarks included: (i) paced QRS duration <140 ms, (ii) precordial transition earlier than lead V4, (iii) absence of QRS notching in inferior leads, and (iv) a q wave, QS, or an isoelectric QRS in lead I.^8^, ^10^ Burri et al., using 3D electroanatomical mapping to validate the ECG criteria for RV septal pacing, found that no single criteria, including a negative QRS in lead I, could accurately distinguish RV mid‐septal pacing from anterior wall pacing.^20^ In the present study, a narrower QRS complex, lesser q waves in the lead I, a more leftward axis, and earlier QRS transitioning in chest leads were suggestive of mid‐septal pacing. Pang et al., who validated the mid‐septal lead position by CT scan, found that the q wave in the lead I was more common with anteroseptal lead position than the septal lead position.^12^ In the study by Burri et al., negative QRS complex or the presence of a q wave in lead I was more common when pacing from the anterior wall compared with from the mid‐septum. This is because of a more leftward position of the lead tip while pacing from the anteroseptal or anterior wall compared with the mid‐septum that has a rightward bulge.^20^ The narrower QRS complex while pacing the mid‐septal region is because of the earlier engagement of the His‐Purkinje system.^21^

According to the fluoroscopic criteria for septal pacing, the RV lead tip should face the spine, with the angle between the horizontal plane and the lead between 0° and 60° in the LAO 40° view. In the RAO 30° view, the lead tip should be in the middle or inferior quadrants.^6^, ^7^, ^10^ Even though the information gained from CT is retrospective, it is more accurate than echocardiography or magnetic resonance imaging for defining the lead tip position and is considered a “gold standard.”^13^, ^22^ In the study by Osmancik et al., mid‐septal lead positioning was achieved in only 41% of patients in whom the LAO 40° criteria for mid‐septum placement were met.^17^ Pang et al.^12^ found that only 21% of the leads were in the true mid‐septum when implanted using conventional fluoroscopic criteria validated by CT.

In a study by Rowe et al., of 10 leads designated to be in the RV septum, seven were on the anterior RV wall, two in the anteroseptal junction, and only one was in the septum on CT scan.^22^ It is now evident from studies using CT scans that conventional fluoroscopy and ECG criteria do not lead to a predictably accurate mid‐septal lead position. During mid‐septal lead implantation, it is most important to avoid implanting the lead in the anterior wall because of the increased risk of left ventricular dysfunction and cardiac perforation.^23^ Studies using CT scans have shown that a significant number of leads implanted using fluoroscopic and ECG landmarks end up on the anterior wall or anteroseptal wall.^22^, ^24^ Inappropriately implanted RV septal leads are associated with poor long‐term LV function and greater dysynchrony than RV apical pacing, which is a significant cause of unreliable and mixed outcomes.^5^, ^18^, ^19^, ^25^, ^26^

RV angiography has been proposed as a guide for accurate mid‐septal lead placement.^12^, ^14^ Shimeno et al.,^15^ using RV angiography in the RAO 30° view as a guide, were able to successfully anchor the RV lead in the septal position in 98% of patients. We believe that a single view does not define the complex RV anatomy. Our study differs from the study by Shimeno et al., as we performed angiography in three views after positioning the lead initially in the presumed septal position and used the angiogram as a guide to reposition the lead. The three angiographic views give a better definition of the 3D RV anatomy than a single RAO view. In the present study, >88% of the leads were in the mid‐septal position, with none in the anterior or lateral walls. Our results show greater accuracy than the study by Shimeno et al., where only 73% of the leads were in the mid‐septum, 23% were in the low septum, and 2% were in the lateral wall. The present study’s particular strength is that we have defined and validated the area of the mid‐septal lead position using an RV angiography‐based grid in three views on the cardiac silhouette. The above‐defined criteria may be considered for mid‐septal lead positioning in future studies. RV angiography can be considered to define the septal anatomy in difficult cases, preferably performed in three views because of the complex RV structure. In the future, the use of preshaped sheaths to deliver the leads accurately to the mid‐septal position can be considered for consistent mid‐septal lead placement instead of manually fashioned dual curve stylet.

### 4.1 Limitations

There are several limitations to our study. First, this was a single‐center study performed on a selected group of patients with normal LV function and AV block. Patients with severe LV dysfunction, valvular heart diseases, or congenital heart diseases were excluded because the dilatation and distortion of the chambers could confound the implant attempts. Another limitation is the small cohort size, which could have led to broader confidence intervals in the statistical analysis. Additionally, in patients in the angiographic group, the lead was repositioned to a better position after reviewing the angiography. This could not be avoided since the purpose of the study was to use angiography to position the lead in the mid‐septal position accurately. We did not compare the ECG between the anterior and septal RVOT pacing, as it was not the study’s purpose and may be a topic of future research. Hemodynamic assessment, pressure–volume loops, and cardiac output were not assessed in the study, either. Since this was a study to determine RV angiography’s utility to define the mid‐septal area and position the lead, we did not assess left ventricular function on follow‐up.

## 5 CONCLUSION

RV angiography in the RAO 30°, LL, and LAO 40° fluoroscopic views define the complex 3D RV anatomy in real‐time during PPM. According to the present study, the appropriate area for mid‐septal lead implantation is the proximal upper segment (segment 1) in the RV angiography‐based 5‐segment grid on the cardiac silhouette in RAO 30°and LL fluoroscopic views. Angiography is a safe and effective method that can be used to confirm the mid‐septal lead position. In difficult anatomical situations or if there is doubt regarding mid‐septal lead positioning during PPM, RV angiography can be used to confirm mid‐septal lead position.

## CONFLICT OF INTEREST

The authors have no conflicts of interest to declare.
